# Design Ultrathin Polyamide Membranes against Funnel Effect: A Novel Zone-of-Influence-Based Approach

**DOI:** 10.1021/acs.est.5c01365

**Published:** 2025-05-16

**Authors:** Yaowen Hu, Pulak Sarkar, Lu Elfa Peng, Fei Wang, Zhe Yang, Chuyang Y. Tang

**Affiliations:** † Department of Civil Engineering, 25809 The University of Hong Kong, Pokfulam, Hong Kong SAR 999077, P. R. China; ‡ Department of Civil and Environmental Engineering, 26680 The Hong Kong Polytechnic University, Hung Hom, Hong Kong SAR 999077, P. R. China; § Dow Centre for Sustainable Engineering Innovation, School of Chemical Engineering, 1974 The University of Queensland, Brisbane, QLD 4072, Australia

**Keywords:** ultrathin membrane, zone of influence, water permeance, membrane design, funnel effect

## Abstract

Ultrathin polyamide membranes have gained significant attention due to their potential to achieve high water permeance. Nevertheless, their water permeance is constrained by the substrate-induced funnel effect. For years, researchers have been investigating how substrates impact membrane water permeance. However, these studies generally rely on a trial-and-error approach to find the optimal substrate porosity, which is often time-consuming and offers limited insights. To establish a more intuitive framework for membrane design, we introduced a novel zone-of-influence (ZOI)-based approach for the first time. We first analyze the distinctively different funnel behaviors for thin and thick films through numerical simulations. Thin films, characterized by small ratios of film thickness over substrate pore size (i.e., aspect ratio θ ≤ 0.5), show a highly localized influence of substrate pores and present a more severe funnel effect than thick films with θ ≫ 1. This analysis leads to the concept of ZOI–a region of polyamide over a single substrate pore with water permeation efficiency exceeding a predefined threshold value. A linear relationship between ZOI and θ was observed, which enables an intuitive design to achieve a target water permeance by simply overlapping ZOIs of multiple pores, making it far more efficient than the traditional trial-and-error approach. We further developed an analytical model based on the superposition principle to unravel the fundamental structure-performance relationship between water permeation efficiency, aspect ratio and substrate porosity. This study provides convenient design tools for optimizing ultrathin membrane structure, offering critical guidance and deep insights for the advancement of high-performance membranes.

## Graphical abstract

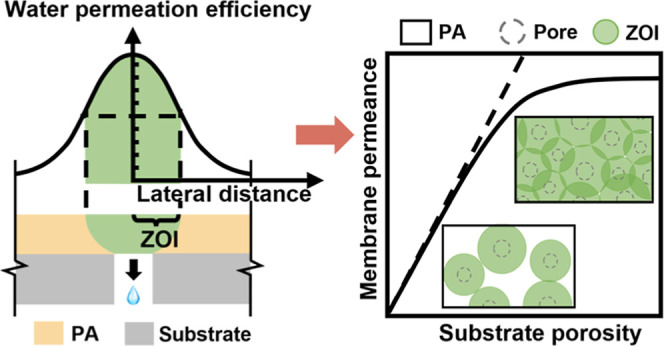

## Introduction

Global freshwater demands have promoted the rapid development of membrane technology,^1–3^ e.g., the wide application of nanofiltration (NF) and reverse osmosis (RO) membranes for replenishment of freshwater by seawater desalination and water reuse.^4–6^ These membranes typically adopt a thin film composite (TFC) configuration with a polyamide (PA) rejection layer on top of a porous substrate.^7–9^ Over the years, massive efforts have been focused on optimizing the PA layer, such as the development of ultrathin rejection layers^10–12^ for enhancing membrane permeance. Whereas the substrate is traditionally regarded as a mere mechanical support,^13–15^ recent literature shows a growing interest in exploring the influence of substrate-induced “funnel effect” on water transport behaviors within membranes.^16–21^

The “funnel effect” refers to a critical transport phenomenon in the PA layer of TFC membranes–water must transport in both lateral and normal directions to reach the pore opening of the substrate.^18,19^ This phenomenon is more pronounced for locations far away from the substrate pore, resulting in overall funnel-shaped pathways that are much longer than the PA layer thickness (see the purple arrows in Figure 1).^16,22^ The funnel effect can severely reduce membrane water permeance, sometimes up to an order of magnitude.^23–25^ Up to date, several modeling studies have suggested that the funnel effect is generally more severe for membranes with a thin PA layer and low substrate porosity.^16,17,22^ These studies generally require prior knowledge of substrate porosity to predict membrane performance. However, from the design point of view, the optimal substrate porosity is often unclear. As a result, current studies typically rely on a trial-and-error approach by trying different parameter settings for substrate optimization, which is time-consuming and lacks fundamental insights. Furthermore, as substantial research efforts are dedicated to decreasing PA thickness,^26–28^ there is an urgent need to excavate the fundamental differences in transport behaviors between thin and thick films.

**Figure 1:**
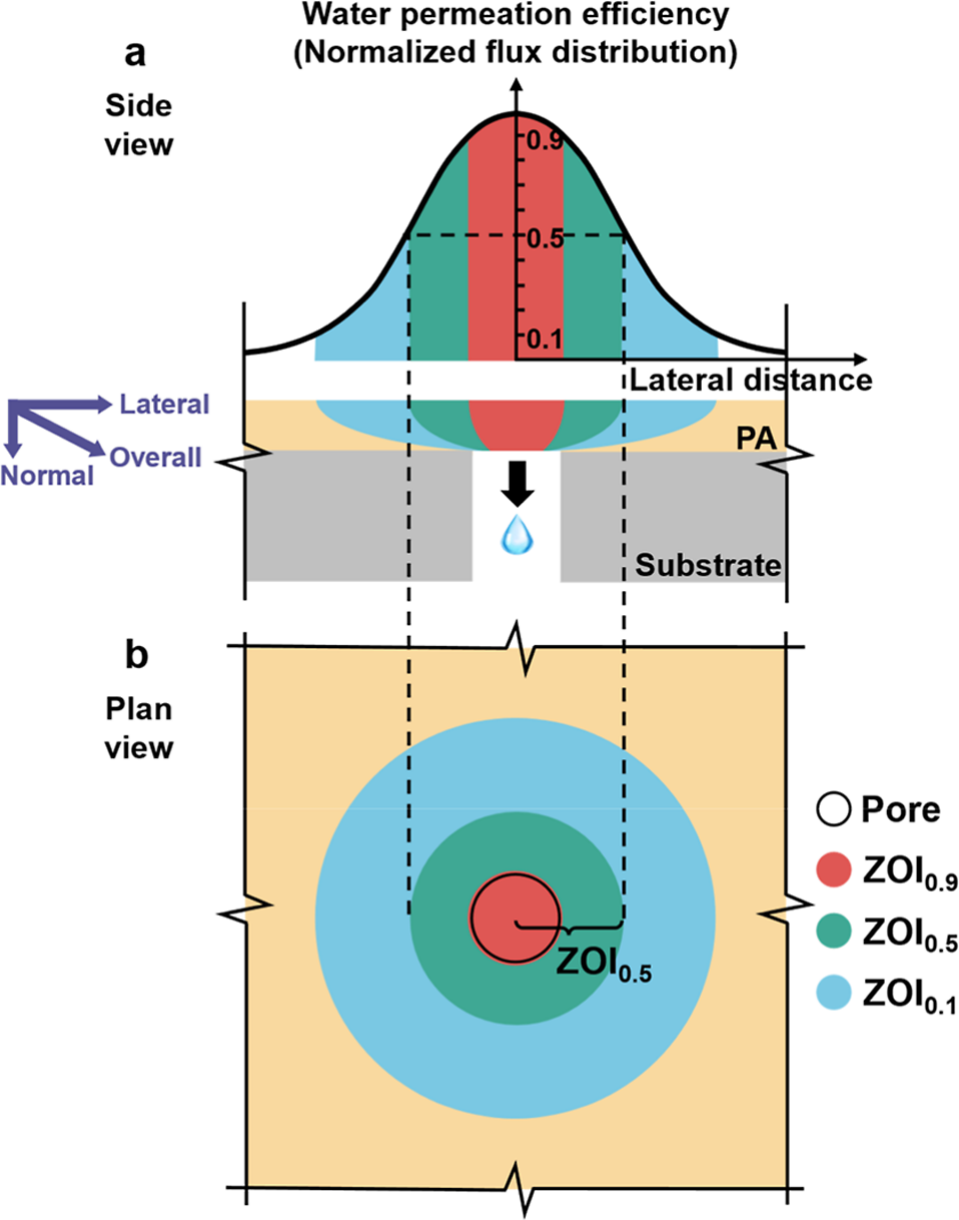
(a) Schematic illustration showing the side view of water permeation efficiency over the membrane surface and funnel-shaped water transport patterns across the PA layer. The water permeation efficiency could be expressed by the water flux normalized by the ideal water flux of a free-standing polyamide film; (b) a plan view showing zones of influence (ZOIs) in different colors corresponding to different water permeation efficiencies. For example, the region colored in green (ZOI_0.5_) represents the area with a water permeation efficiency of at least 0.5. The PA layer spans over a single substrate pore and is assumed to be infinitely large in the lateral direction.

Much can be learned from the concept of zone of inhibition in microbiology, which assesses the effectiveness of an antimicrobial agent by measuring the zone where the bacteria cannot grow around the agent.^29,30^ Inspired by this concept, we can similarly define the zone of influence (ZOI) of a single substrate pore to reflect its effectiveness for water permeation. Specifically, based on the distribution of water permeation efficiency adjacent to an individual substrate pore, ZOI refers to the PA surface where the efficiency exceeds a specified threshold value (e.g., ZOI_0.5_ with a threshold value of 0.5 in Figure 1). The quantification of ZOI allows us to develop a rational approach for intuitive design of substrate pore distributions to achieve a desired overall water permeation efficiency. To more rigorously measure the cumulative effects of multiple pores on water permeation, we hypothesize that the influence of multiple pores can be obtained by superimposing those of individual pores. Based on this superposition approach, we further establish an analytical model to decipher the fundamental membrane structure-performance relationship. Our study offers deep insights into membrane water transport mechanisms and provides a convenient tool for the design of ultrathin membranes.

## Model Development

### Numerical Simulations

In this study, we conducted three-dimensional (3D) simulations to analyze the transport of water through the PA layer, generally following procedures reported in our earlier studies.^24^ Briefly, the steady-state water transport in the PA layer can be described by the Darcy’s law (**J**_*w*_ = −*P*_*w*_^*H*^∇*p* ^*t*^), which relates the water flux vector (**J**_*w*_) to the net driving force (*p* ^*t*^) and the hydraulic permeability (*P*_w_^*H*^).^31,32^ By further combining with the mass conservation principle (∇·**J**_*w*_ = 0), the governing equation is specified in the form of the Laplace equation:

1 $$\nabla^{2}p^{t}=0$$

In eq 1, the net driving pressure *p* ^*t*^ can be obtained by subtracting the osmotic pressure from the applied hydraulic pressure (i.e., *p* ^*t*^ = *p*–π). The symbol ∇ denotes a three-dimensional vector operator in this study (i.e., $\frac{\partial }{\partial x},\frac{\partial }{\partial y},\frac{\partial }{\partial z}$ ). The governing equation (eq 1), together with the boundary conditions implicated in Figure S1 (see Supporting Information Section S1), can be numerically solved by the finite element method in the commercial software COMSOL Multi-Physics (v5.4). Figure S2 further validates the numerical model by showing a strong correlation between the experimentally obtained water permeability^33^ and the simulated water permeation efficiency.

### Analysis of Unit Membrane Cell

In this study, to investigate how an individual pore contributes to overall membrane performance, we consider a unit membrane cell that has infinite lateral dimensions and contains a single substrate pore. To implement this in COMSOL simulations, the lateral size (*R*_*c*_) adopted in the simulations needs to be sufficiently large compared to the pore size (*R*_*p*_). Based on the detailed analysis in Figure S3, a *R*_*c*_/*R*_*p*_ ratio of 20 is large enough to approximate an infinite membrane cell, as this ratio has a negligible impact on simulation results once exceeding 20. In this study, a further normalization of the film thickness δ by the substrate pore size *R*_*p*_ is helpful to simplify the problem:

2 $$\theta =\frac{\delta }{R_{p}}$$

where θ is the aspect ratio. As Figure S4 shows, one could find similar membrane performance for the same θ value.

## Results and Discussion

### Funnel Effect and the Role of Aspect Ratio on Water Transport

In recent years, there has been tremendous interest in reducing the thickness of PA rejection layer in order to enhance membrane water permeance.^34–36^ Figure 2 shows the role of PA thickness (δ) on water transport patterns for a PA layer spanning over a single pore with a fixed diameter (*R*_*p*_ = 10 nm). Regardless of the PA thickness, the water transport follows curved pathways that resemble a funnel-like shape–the origin of the so-called funnel effect. Nevertheless, the specific shape of these funnels strongly depends on the aspect ratio (θ = δ/*R*_*p*_) of the membrane. For a relatively thin PA layer of 5 nm (θ = 0.5), the water transport streamlines are highly concentrated near the pore region, with nearly straight streamlines along the normal direction (Figure 2a). As a result, the local water flux near the pore center is close to the ideal flux (*J*_ideal_), and it dramatically decreases over a narrow funnel-shaped transition region near the pore edge. Figure 3 further plots the distribution of normalized local flux (*J*_local_/*J*_ideal_) over the membrane surface. For the membrane featuring θ = 0.5 (Figure 3b), this normalized value transits from 0.9 to 0.1 within a narrow window (*x*/*R*_*p*_ = 0.69 to 1.66). This window of transition becomes even narrower (*x/R*_*p*_ = 0.95 to 1.14) for a thinner membrane with a θ value of 0.1 (Figure 3a).

**Figure 2:**
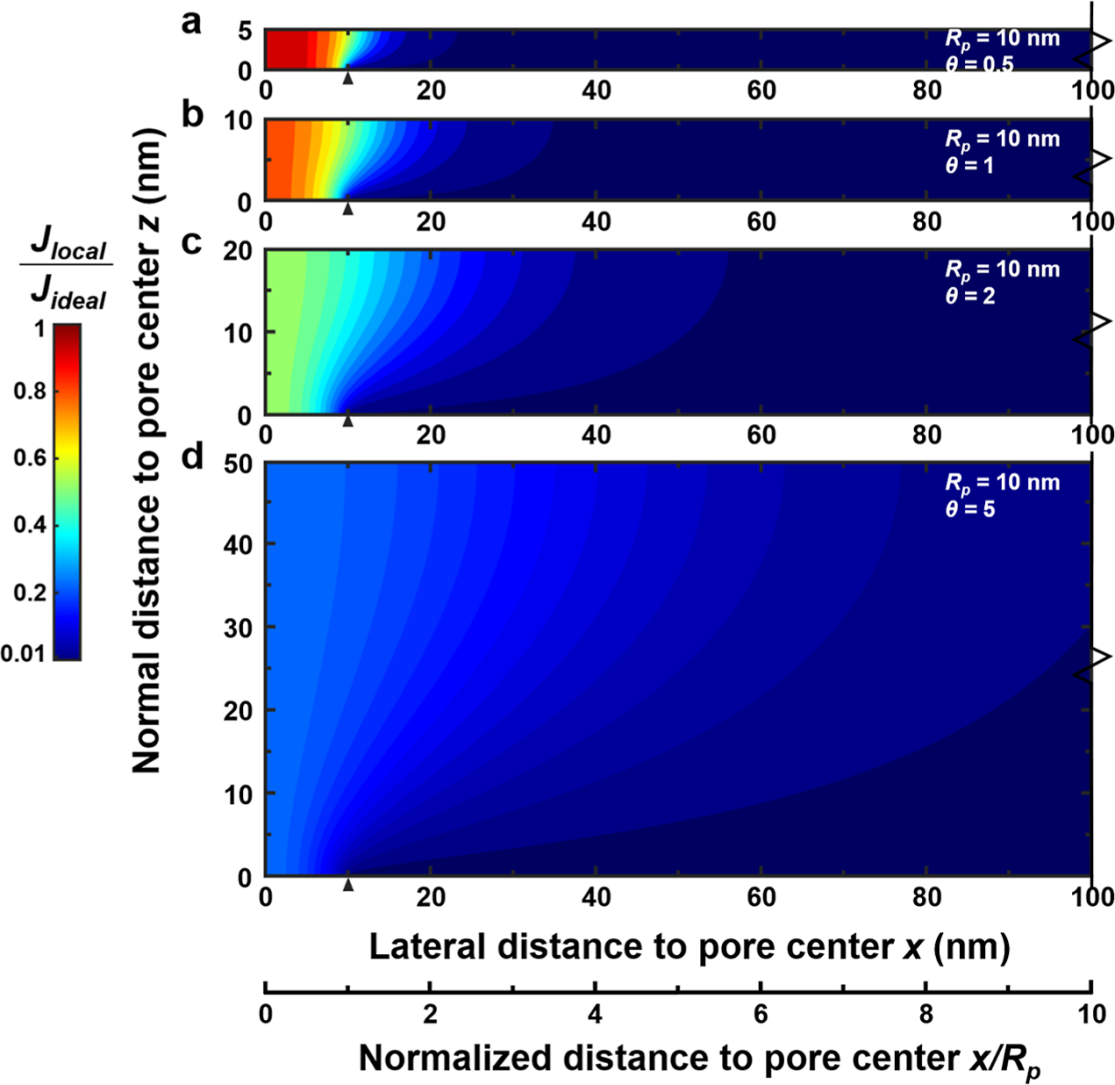
Water transport patterns across the PA layer for a unit TFC membrane cell with infinite lateral dimensions and a single substrate pore. The PA thickness (δ) varies from 5 to 50 nm, and the substrate pore size (*R*_*p*_) is fixed at 10 nm. The location of *x* = 0 represents the substrate pore center, and the location of *x* = 10 nm represents the pore edge (indicated by the black triangle).

**Figure 3:**
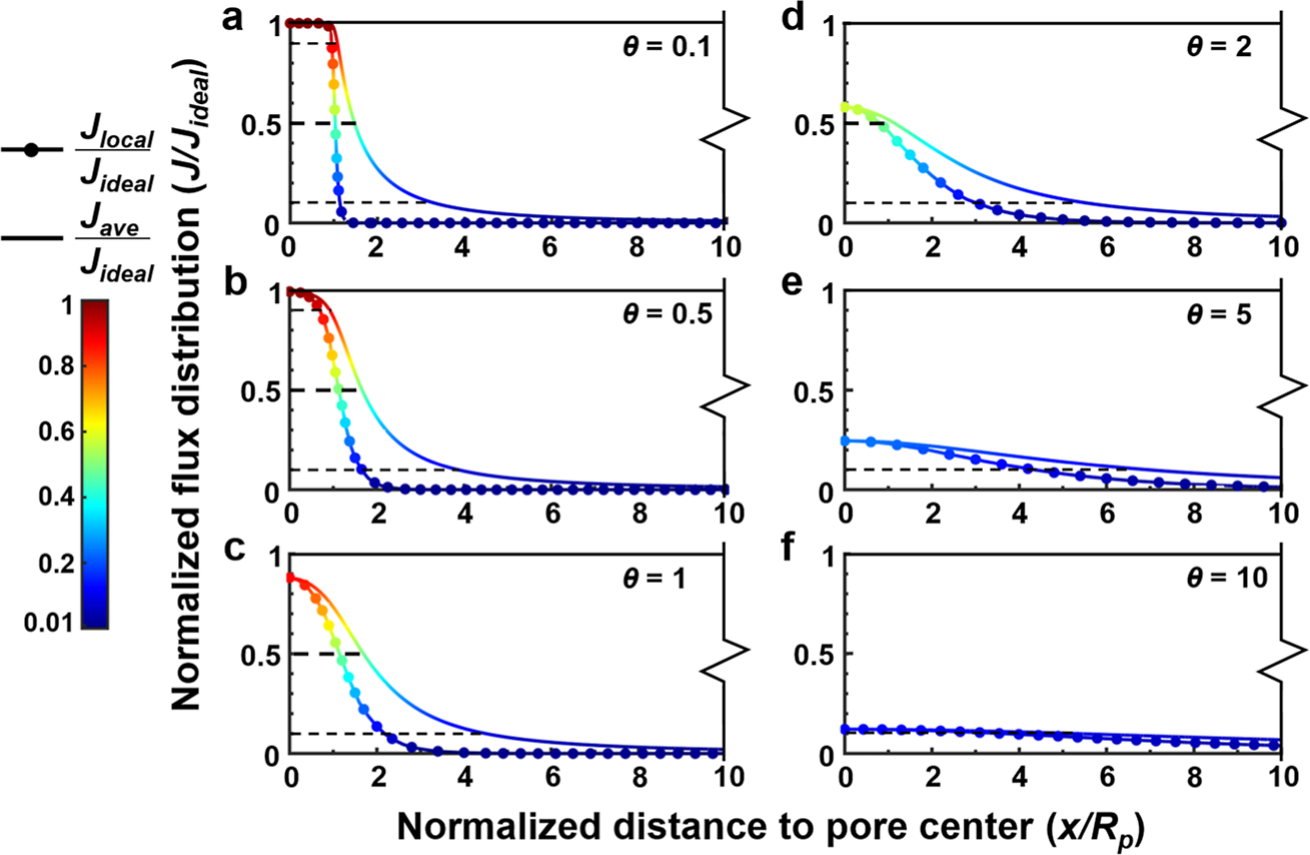
Flux distributions over the top membrane surface for a unit TFC membrane cell with infinite lateral dimensions and a single substrate pore. Membranes with different aspect ratios (θ) and a fixed substrate pore size (*R*_*p*_ = 10 nm) are simulated. The curves with circular symbols represent the local water flux normalized by the ideal water flux of a free-standing polyamide film, while the smooth curves without symbols refer to the area-averaged water flux normalized by the ideal water flux. The black dashed lines show the locations where the normalized water flux is equal to 0.1, 0.5, and 0.9, respectively.

Thick membranes exhibit considerably different water transport behavior. At a high θ value of 5, water transport streamlines tend to spread out more, resulting in fatter funnels that can collect water from a wider PA area (Figure 2d). As a result, the normalized local flux (*J*_local_/*J*_ideal_) gradually decreases with distance from the pore center (Figure 3e). The rate of decrease is slower when the film thickness further increases (Figure 3f). At the same time, the maximum local water flux (*J*_max_) at the pore center is only about 0.25*J*_ideal_ for θ = 5 (Figure 3e) and 0.12*J*_ideal_ for θ = 10 (Figure 3f). For these membranes, the water streamlines enter the film from a wide region but greatly concentrate near the pore center when exiting the film, causing a major reduction in the available cross-sectional area (Figure S5) and thus an increased hydraulic resistance. Notably, both Figures 2 and 3 report the local water flux normalized by the ideal flux (*J*_local_/*J*_ideal_). The effective local water flux (*J*_local_) of thick membranes could be further smaller than that of thin membranes since the ideal flux (*J*_ideal_) is inversely proportional to PA thickness.

Although the above discussions are based on different PA thicknesses at a fixed substrate pore size (*R*_*p*_ = 10 nm), similar membrane transport behaviors could be obtained by varying substrate pore sizes at a constant PA layer thickness (δ = 20 nm, Figure S6). Enlarging substrate pore size shows a similar effect on the funnel shape with decreasing film thickness. Indeed, membranes with the same aspect ratio θ present the same water transport patterns regardless of their PA thicknesses and pore sizes (Figures S6 vs [Fig fig2]), which is further confirmed by the analysis in Figure S4. These results highlight the critical importance of the aspect ratio θ in determining the funnel behavior. This ratio could additionally serve as a fundamental parameter to define thin and thick films: thin films are characterized by aspect ratios much lower than 1 (e.g., θ ≤ 0.5) whereas thick films have much higher aspect ratios (θ ≫ 1). These two cases exhibit distinct membrane flux behaviors. For thin films with θ ≤ 0.5, the normalized local flux (*J*_local_/*J*_ideal_) approaches 1 at the pore center, ∼0.7 at the pore edge, and 0 away from the pore edge (Figure S7a). In contrast, when θ ≫ 1, *J*_local_/*J*_ideal_ decreases with increasing θ at both the pore center and the pore edge, yet it increases with increasing θ away from the pore edge. As a result, the contribution of the flow directly above the pore region (*q*_*p*_) to the total water permeation of a single pore (*q*_*T*_) is significant for thin films (θ ≤ 0.5) but tends to be far less important for thick films (θ ≫ 1) (Figure S7b). In addition, it is interesting to note that, regardless of the specific θ value, the flux distributions of thin films (θ ≤ 0.5) almost overlap by plotting *J*_local_/*J*_max_ against the normalized distance to the pore edge ((*x*-*R*_*p*_)/δ). Similarly, thick films also exhibit nearly identical distributions of *J*_local_/*J*_max_ with respect to the pore edge as long as θ ≫ 1 (Figure S7c). This observation provides a convenient way to predict flux distributions of thin and thick films.

### ZOI Concept

Figure 3 also allows us to introduce the ZOI concept to account for the extent to which a single pore affects the localized water transport. Specifically, ZOI is defined as the region where the localized water permeation efficiency (*J*_local_/*J*_ideal_) exceeds a certain threshold value (ξ, ranging from 0 to 1). For example, considering a threshold value (ξ) of 0.5, ZOI_0.5_ indicates the region where the localized water permeation efficiency exceeds 0.5 (*J*_local_/*J*_ideal_ ≥ 0.5). This corresponds to a circular region over the membrane top surface due to the axisymmetric feature of the membrane cell (see the schematic diagram in Figure 1b). For a thin film with an aspect ratio θ of 0.1, the radius of ZOI_0.5_ corresponds to a normalized lateral distance *x/R*_*p*_ = 1.03 (Figure 3a). This value changes as θ varies (*x/R*_*p*_ = 1.13, 1.14, and 0.81, for θ = 0.5, 1 and 2, respectively, Figure 3b–d). Similar to ZOI_0.5_, we can define ZOI based on other threshold values (e.g., ZOI_0.1_ corresponding to ξ = 0.1 and ZOI_0.9_ corresponding to ξ = 0.9). It is worthwhile to note that ZOI with high threshold values ξ may not be attained for thick membranes whose maximum flux is relatively low. For example, in Figure 3e (θ = 5), one can define ZOI_0.1_ but not ZOI_0.9_ or ZOI_0.5_. Figure 3 also presents the normalized area-averaged water flux (*J*_ave_/*J*_ideal_), which allows us to define the average flux-based zone of influence ( $\overset{¯}{\mathrm{ZOI}}$ ). Specifically, $\overset{¯}{\mathrm{ZOI}}$ refers to the PA region where *J*_ave_/*J*_ideal_ exceeds a predefined threshold value ξ̅. The concept of ZOI provides a useful tool for membrane design and more details will be discussed in the next section.

### Effect of Aspect Ratio on ZOI

Figure S8 shows the effect of aspect ratio over a wide range (0.1 ≤ θ ≤ 10) on ZOI. Generally speaking, ZOI will first widen and then narrow as the aspect ratio θ increases. Due to the growing interest in reducing PA thickness among the research community,^36^ we mainly focus on thin films in this study. As shown in Figure 4a, thin films exhibit a linear dependence of the localized ZOI on θ (for 0 < θ ≤ 0.5). For each threshold value, a characteristic slant angle (ϕ) can be obtained. This angle is uniquely determined by the threshold value ξ of ZOI (e.g., ϕ = 76° for ξ = 0.5 in Figure 4a,b). For design purposes, one can obtain ϕ from Figure 4b, which allows us to conveniently determine ZOI by simply drawing a straight line from the pore edge with this slant angle to intersect with the PA top surface (e.g., the dotted lines in Figure 4c) for various combinations of ξ and θ values. For example, by plotting a line with ϕ = 76° (the green line in Figure 4c), the radius of ZOI_0.5_ (the gray lines in Figure 4c) that corresponds to different θ values can be determined.

**Figure 4:**
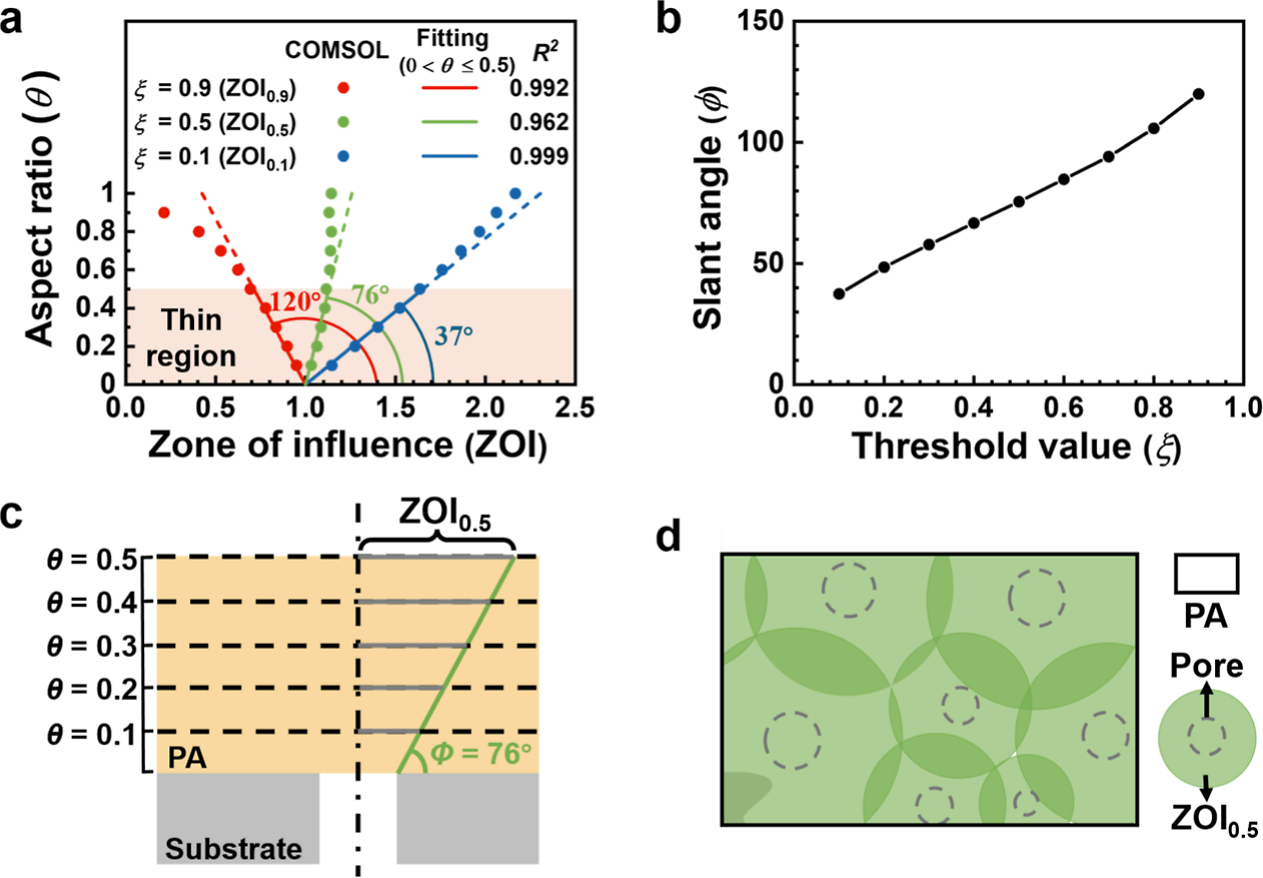
(a) The effect of aspect ratio θ on the localized ZOI considering three convenient threshold values ξ of 0.1, 0.5, and 0.9 (i.e., ZOI_0.1_, ZOI_0.5_, and ZOI_0.9_). For a given ξ value, the ZOI is linearly dependent on θ for 0 < θ ≤ 0.5, with *R* ^2^ values greater than 0.95. Assuming the linear fitting lines pass through the pore edge (*x/R*_*p*_ = 1), their characteristic slant angles ϕ are about 37°, 76°, and 120°, respectively. (b) The correlation between the linear fitting angle ϕ and the threshold value of water permeation efficiency ξ for thin films featuring θ ≤ 0.5. Membranes with different PA thicknesses (δ) and a fixed substrate pore size (*R*_*p*_ = 10 nm) are simulated. (c) Schematic illustration of how to apply the characteristic slant angle ϕ to get the corresponding ZOI for membranes featuring different θ values. Their top surfaces are indicated by the dashed lines. (d) Schematic diagram of a membrane cell (black square) covered by ZOI_0.5_ (shaded green circles) of different substrate pores (dashed gray circles).

### Intuitive Membrane Design Based on ZOI

Here, we establish a convenient framework to design TFC membranes on the basis of ZOI (Figure 4d). In practical applications, a target water permeance is often specified (e.g., >50 Lm^2–^ h^–1^ bar^–1^ for vacuum-driven nanofiltration^37^). By measuring the PA layer thickness, one could determine the ideal water permeance and thus the target minimal water permeation efficiency ξ. For example, to achieve a minimum water permeation efficiency of 0.5 for a substrate containing multiple pores (i.e., ξ = 0.5), we can obtain ZOI_0.5_ (shaded green circles) of each substrate pore (dashed gray circles) using Figure 4b,c. We then tailor the substrate pore distribution until complete coverage of the PA surface by the overlapping ZOI_0.5_ regions. Notably, while increasing substrate porosity could enhance water permeation efficiency, excessively high porosity may compromise membrane mechanical strength, rendering membranes susceptible to compaction and structural failure.^38,39^ It should be noted that the average water flux (*J*_ave_/*J*_ideal_) and average zone of influence ( $\overset{¯}{\mathrm{ZOI}}$ ) are also of great practical significance since these parameters are more directly linked to the overall membrane water permeance. Similar to the analysis of ZOI on the basis of localized flux, we can obtain the characteristic slant angles ϕ̅ for $\overset{¯}{\mathrm{ZOI}}$ defined based on the average flux (Figure S9). This allows us to identify the $\overset{¯}{\mathrm{ZOI}}$ region of different substrate pores by using the ϕ̅ angle and tailor substrate pore distribution to achieve a target minimum overall water permeance efficiency.

### Analytical Membrane Design Based on the Superposition Principle

In addition to the intuitive design based on ZOI, this study also proposes a more sophisticated analytical design method based on the assumption that the influence of different individual pores can be superimposed. To test this superposition hypothesis, we simulated the local flux distribution for a substrate containing two pores (the blue dotted line in Figure 5). At the same time, their superimposed influence (the red solid line) was obtained by simply adding the local fluxes separately simulated for each individual pore (the purple dotted lines). As shown in the inset of Figure 5, the superimposed results generally agree well with the simulation for the double-pore case, despite some slight deviations when the two pores become very close. Furthermore, we obtained the total flow rate of a double-pore system via two approaches: (1) by numerically integrating the simulated local flux over the membrane surface (*Q*_*T*_); and (2) by multiplying the flow rate of each pore (*q*_*T*_) by the number of pores (*N* = 2). Figure S10a shows that the superimposed *Nq*_*T*_ agrees well with the simulated *Q*_*T*_ for thin films (θ ≤ 0.5), with an error of less than 2% even for a very close pore-to-pore distance (*D*/*R*_*p*_ = 1.2). Figure S10b further extends the superposition principle to thin films containing more substrate pores. For all cases, the superposition principle shows reasonable accuracy for thin films.

**Figure 5:**
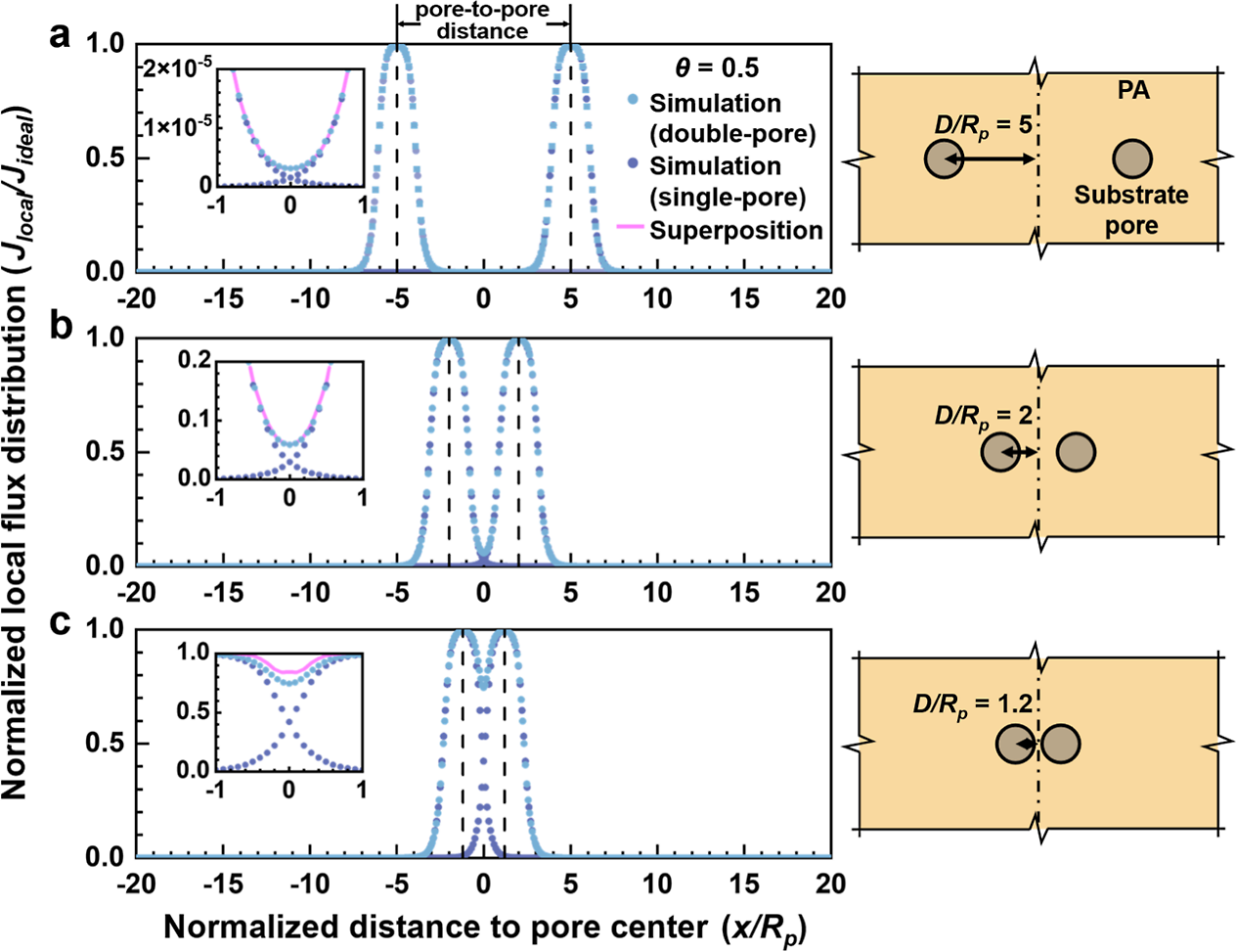
Normalized local flux distributions (*J*_local_/*J*_ideal_) in the presence of two substrate pores (the blue dotted line). The normalized flux distribution resulting from each individual substrate pore is also simulated (the purple dotted line). The red solid line is obtained by the superposition of the two purple dotted lines. The schematic drawings on the right illustrate the change in the pore-to-pore distance (with *D* representing half of the pore-to-pore distance). Other simulation conditions include: the substrate pore size *R*_*p*_ = 10 nm and film thickness δ = 5 nm.

The superposition principle allows one to estimate the total water flow rate of a multipore system from that of individual pores as follows:

3a $$Q_{T}=N\cdot q_{T}$$

where *q*_*T*_ of an individual pore can be obtained by

3b $$q_{T}=\gamma \cdot S_{p}\cdot J_{ideal}$$

In eq 3b, *J*_ideal_ is the ideal water flux corresponding to a free-standing polyamide film and *S*_*p*_ is the area of the substrate pore. As a result, their product (*q*_ideal_ *= S*_*p*_·*J*_ideal_) can be interpreted as an ideal flow rate over the pore region. Typically, the actual available flow rate (*q*_*T*_) will be greater than the ideal flow rate (*q*_ideal_) due to the additional contribution from the region adjacent to the pore (Figure 6). To quantify this effect, we introduce a flow rate enhancement factor γ in eq 3b. Interestingly, Figure 6 shows a linear dependence of γ on the aspect ratio θ for thin films. This linearity, combined with the superposition principle, enables the estimation of the water permeation efficiency (*A*_app_/*A*_ideal_) of a porous membrane (see detailed derivations in Supporting Information S11):

4 $$\frac{A_{app}}{A_{ideal}}=\gamma \cdot \varepsilon ,(\mathrm{for}\;\gamma \cdot \varepsilon <1)$$

where ε is the substrate porosity, *A*_app_ is the apparent water permeance of a substrate-supported polyamide membrane, and *A*_ideal_ is the ideal water permeance of a free-standing polyamide film. The sensitivity analysis (Figure S11) shows a good agreement between the analytical model and COMSOL simulations. For design purposes, to achieve a target water permeation efficiency (e.g., *A*_app_/*A*_ideal_ = 0.5) for a thin film featuring a specific θ value, one can use Figure 6 to obtain the corresponding γ value and thus determine the desired porosity (ε = 0.5/γ) according to eq 4.

**Figure 6:**
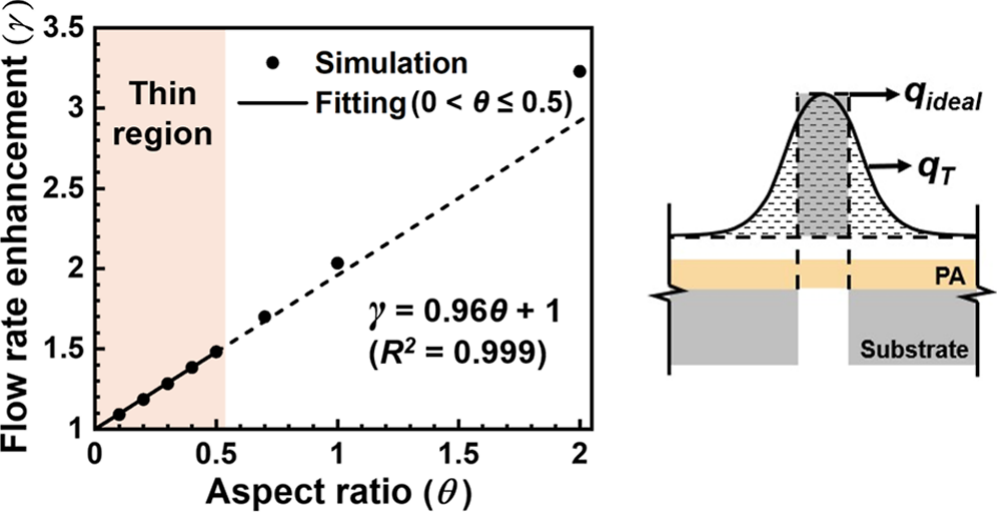
Effect of aspect ratio (θ) on the flow rate enhancement (γ *= q*_*T*_/*q*_ideal_), where *q*_*T*_ is the total flow rate obtained from simulation (the patterned region of the schematical drawing). The simulated membrane cells are assumed to be infinitely large in the lateral dimension and contain a single substrate pore. The ideal flow rate *q*_ideal_ is the product of the ideal flux and the area of the pore (the gray-colored region of the schematical drawing). The linear fitting line is obtained by fitting data over 0 < θ ≤ 0.5.

To provide further mechanistic insights on membrane transport, we plot four lines of fundamental significance in Figure 7. Line 1 represents the ideal water permeance of a free-standing polyamide film (*A*_ideal_), assuming all PA regions contribute to water permeation without suffering substrate-induced funnel effect. Therefore, *A*_ideal_, not affected by the substrate porosity, sets an upper limit for the available water permeance. In contrast, Line 2, the substrate-limited water permeance (ε·*A*_ideal_) that only considers the contribution from the PA region directly above the pore, could be regarded as the lower limit of water permeance. The actual apparent water permeance (*A*_app_, Line 3) of a substrate-supported membrane is bounded by Line 1 and Line 2, with contributions from both the pore region and the adjacent shoulder region. In Figure 7, we also present Line 4 (γ·ε), which is derived from the superposition principle (eq 4).

**Figure 7:**
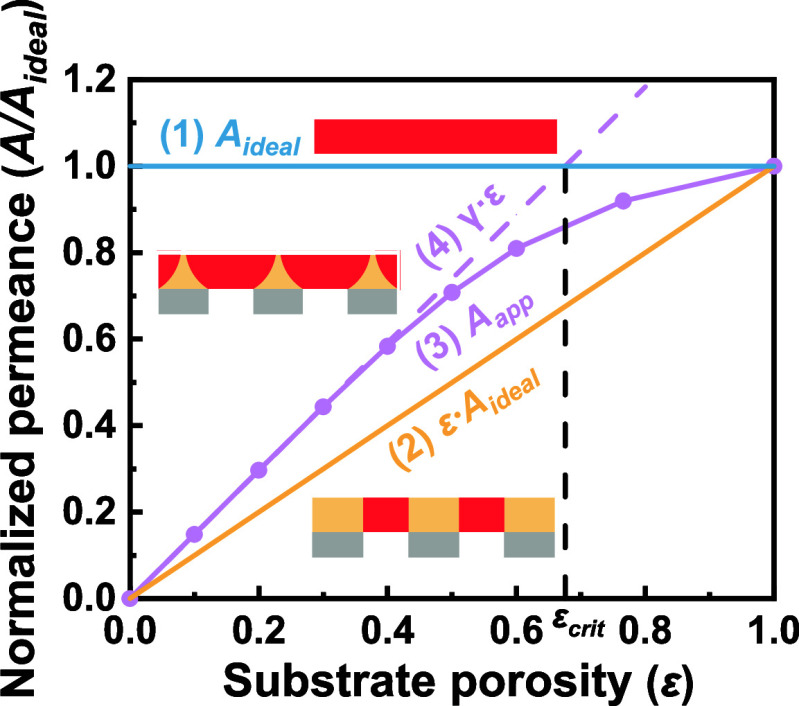
Graphical illustration of normalized water permeance of ultrathin polyamide membranes. Line 1 represents the ideal water permeance of a free-standing polyamide film which could collect water from all PA regions. Line 2 represents the substrate-limited water permeance, assuming that only the PA region directly above the pore could contribute to water permeation. Line 3 represents the apparent water permeance of a substrate-supported membrane, considering the contributions from both the pore region and the adjacent shoulder region. In Line 3, the simulated membrane cells adopt a θ value of 0.5. Line 4 is a linear straight line with a slope of γ as derived from eq 4.

Interestingly, Line 4 shows an initial linear part that nearly coincides with Line 3. When the substrate porosity increases, Line 4 will eventually deviate with Line 3 due to the severe overlapping of ZOIs of substrate pores. The intersection of Line 4 and Line 1 defines a critical porosity (ε_crit_):

5 $$\varepsilon_{crit}=1/\gamma$$

When the actual porosity ε exceeds ε_crit_, a further increase in ε becomes less effective for enhancing water permeance and Line 3 gradually approaches the ideal water permeance (Line 1). Therefore, it is of little practical value to aim for an excessively high substrate porosity (ε > ε_crit_). Our study reveals the important yet complex role of substrate porosity on water transport, calling for more systematic further investigations on substrate design and optimization.

## Environmental Implications

In recent years, researchers have shown a growing interest in optimizing ultrathin polyamide membranes to achieve high water permeance, which holds great potential for reducing energy consumption, particularly for applications involving low-osmotic-pressure feed solutions.^26,40^ Furthermore, highly permeable membranes may enable innovative module and process design such as submerged vacuum-driven nanofiltration.^26,34^

In the current study, we developed convenient tools for ultrathin membrane design based on the novel ZOI concept. This ZOI-based approach offers a unique perspective by investigating the effectiveness of a single substrate pore for water collection, whose contribution to the overall water permeation can be conveniently obtained by superposition. This makes the ZOI-based approach more accessible over those traditional trial-and-error and computationally extensive methods. Notably, our study reveals a strong linear correlation between θ and γ for thin films with θ ≤ 0.5 (Figure 6). Extending this correlation to θ = 1 resulted in a slight deviation of approximately 4%. However, the linear correlation is no longer valid for thick films with θ > 1. Nevertheless, the ZOI-based approach can still be extended to thick films by using Figure S8 to determine ZOI of different substrate pores.

Even though our work has primarily focused on the design for polyamide membranes in the context of water treatment, the novel ZOI-based approach can be easily extended to other membrane types such as gas separation and pervaporation.^24,28,35,41^ Moreover, beyond water transport, future studies need to further explore the transport of solutes through membranes to provide a more comprehensive understanding of membrane separation performance. Additionally, this study captures key structure parameters and employs an idealized flat PA layer over the substrate. Future studies may further consider more complicated membrane structures such as nanoscale porosity of PA, its structural heterogeneity and its intrusion into substrate pores.

## Supplementary Material



## Notes

Boundary condition settings for COMSOL simulations (Section S1); experimental validation (Section S2); sensitivity test of *R*_*c*_/*R*_*p*_ ratio (Section S3); normalization processing of membrane structural parameters (Section S4); water transport streamlines in the PA layer (Section S5); Effect of substrate pore size on water transport patterns (Section S6); flux behaviors of thin and thick membranes (Section S7); effect of aspect ratio on zone of influence (Section S8); characteristic slant angles based on the average zone of influence (Section S9); accuracy test of superposition principle (Section S10); total flow rate calculation of a multipore system (Section S11); and sensitivity analysis of a multipore system (Section S12) (PDF)
