# Supplementary material for: Design Ultrathin Polyamide Membranes against Funnel Effect: A Novel Zone-of-Influence-Based Approach
Source: Environ Sci Technol. 2025 May 16;59(21):10600–7. doi: 10.1021/acs.est.5c01365 (PMC12138970; doi:10.1021/acs.est.5c01365)
Supplement: Supplementary file 1 [file es5c01365_si_001.pdf]

# Supporting Information

# Design Ultrathin Polyamide Membranes against Funnel Effect: A Novel Zone-of-influence-based Approach

Yaowen Hu,[1] Pulak Sarkar,[1] Lu Elfa Peng,[1] Fei Wang,[2] * Zhe Yang,[1 3] and Chuyang Y. Tang[1] *

[1] Department of Civil Engineering, the University of Hong Kong, Pokfulam, Hong Kong, SAR 999077, P. R. China

[2] Department of Civil and Environmental Engineering, The Hong Kong Polytechnic University, Hung Hom, Hong Kong, SAR 999077, P. R. China

[3] Dow Centre for Sustainable Engineering Innovation, School of Chemical Engineering, The University of Queensland, Brisbane, QLD 4072, Australia

* Corresponding Author

Phone: +852 2859 1976, E-mail address: tangc@hku.hk

Phone: +852 6581 2701, E-mail address: felixwf.wang@polyu.edu.hk

Number of pages: 17

Number of figures: 11

**CONTENTS**

**S1. Boundary condition settings for COMSOL simulations (Page S3)**

**S2. Experimental validation (Page S4)**

**S3. Sensitivity test of $R_c/R_p$ ratio (Page S5)**

**S4. Normalization processing of membrane structural parameters (Page S6)**

**S5. Water transport streamlines in the PA layer (Page S7)**

**S6. Effect of substrate pore size on water transport patterns (Page S9)**

**S7. Flux behaviors of thin and thick membranes (Page S10)**

**S8. Effect of aspect ratio on zone of influence (Page S12)**

**S9. Characteristic slant angles based on the average zone of influence (Page S13)**

**S10. Accuracy test of superposition principle (Page S14)**

**S11. Total flow rate calculation of a multi-pore system (Page S15)**

**S12. Sensitivity analysis of a multi-pore system (Page S16)**

## S1. Boundary condition settings for COMSOL simulations

**Figure S1** schematically illustrates the 3D geometry of an infinite TFC membrane cell, with boundary conditions used for COMSOL simulations included. In view of the impermeable feature of the substrate material, the solid fraction of the substrate is assigned to a zero-flux condition. The net driving pressure $p^t$ is subject to further normalization ($\hat{p}^t = \frac{p^t - p_p^t}{p_f^t - p_p^t}$), where $\hat{p}^t$ is the normalized total driving pressure, and $p_f^t$ and $p_p^t$ are the total driving pressures at the feed and permeate sides, respectively. The feed/film and film/pore interfaces have $\hat{p}^t$ values of 1 and 0, respectively. Flux continuity is imposed throughout the PA layer.

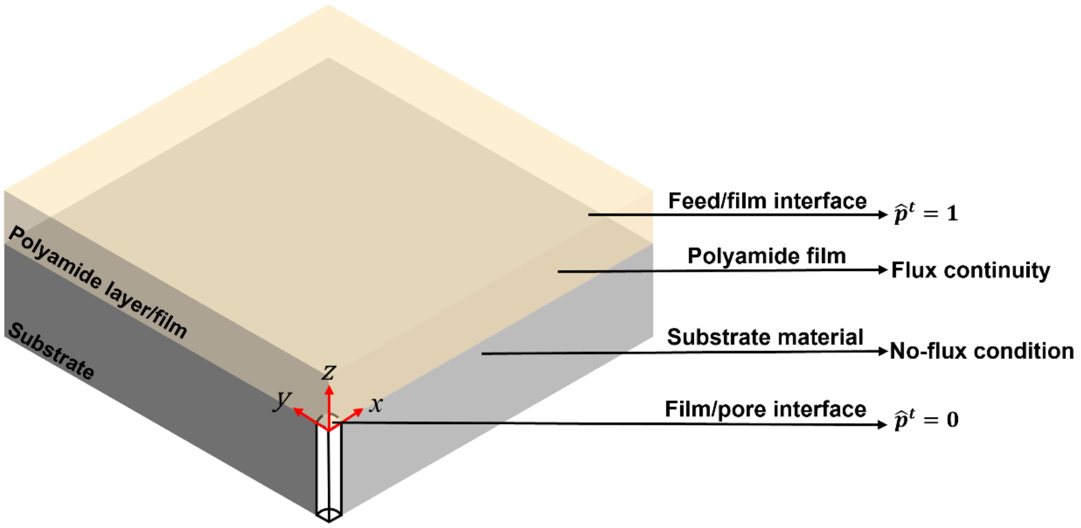


**Figure S1.** Summary of boundary conditions for COMSOL simulations of water transport in a 3D infinite TFC membrane cell.

## S2. Experimental validation

We used the experimental data from Jiang et al.[1] to validate the numerical model. These authors prepared polyamide films over a wide range of thickness values (4.2–29.8 nm). According to them, the average pore size of the substrate is 15.5 nm. As the substrate porosity is not reported in their study, we converted the SEM image of the PSF support into a binary black and white format (**Figure S2a**). A porosity of 10.7% was obtained by analyzing the binary image using Image-Pro Plus (Media Cybernetics, Inc.). In general, the simulated water permeation efficiency shows a strong linear correlation with experimentally observed water permeability (**Figure S2b**). These results demonstrated that the numerical model is capable of predicting water transport behavior.

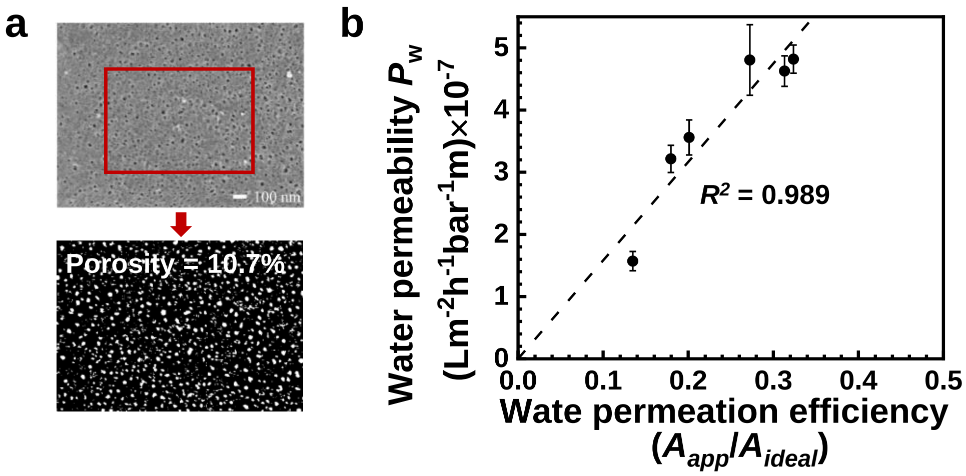


**Figure S2.** (a) Conversion of substrate SEM image into a binary format to calculate substrate porosity. The upper panel is reproduced from ref 1 with permission (Copyright 2020 American Chemical Society). (b) The correlation between the simulated water permeation efficiency and the experimentally obtained water permeability.

## S3. Sensitivity test of $R_c/R_p$ ratio

To determine a proper cell size to pore size ratio ($R_c/R_p$) for approximating membrane cells that extend infinitely in the lateral direction, we investigate the impact of $R_c/R_p$ ratio on the normalized local flux distributions. As shown in **Figure S3**, regardless of the value of $\delta/R_p$, the flux distributions for $R_c/R_p$ ratios of 20 and 40 almost overlap. To reduce computational load, this study adopts a $R_c/R_p$ ratio of 20 to approximate the infinite lateral dimension of a membrane cell since no major variation of the simulation results is found when this ratio further increases.

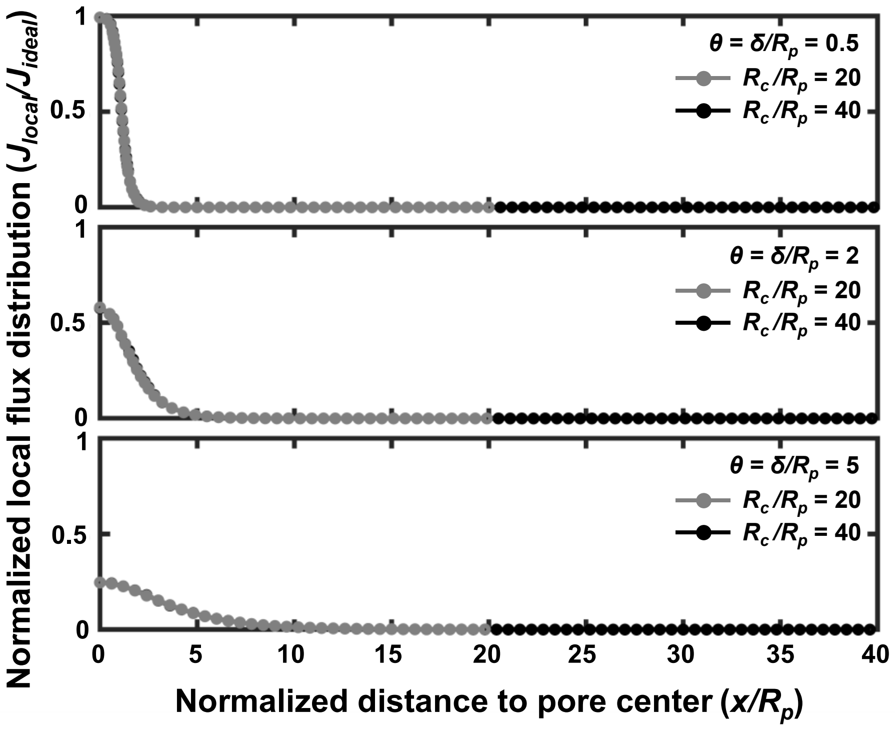


**Figure S3.** The impact of the $R_c/R_p$ ratio on the normalized local flux distributions over the membrane surface. The PA thickness ($\delta$) varies from 5 nm to 50 nm, and the substrate pore size ($R_p$) is fixed at 10 nm.

## S4. Normalization processing of membrane structural parameters

**Figure S4a** plots local water flux distributions ($J_{local}$) as a function of the lateral distance to pore center ($x$) for membranes with different $\delta$ and $R_p$ values but an identical $\theta$ value of 0.5. When taking the normalized local water flux as the vertical axis ($J_{local}/J_{ideal}$) and the normalized distance to the pore center ($x/R_p$) as the horizontal axis in **Figure S4b**, the two curves coincide completely with each other. This also happens for membranes with a high $\theta$ value of 5 (**Figure S4c,d**).

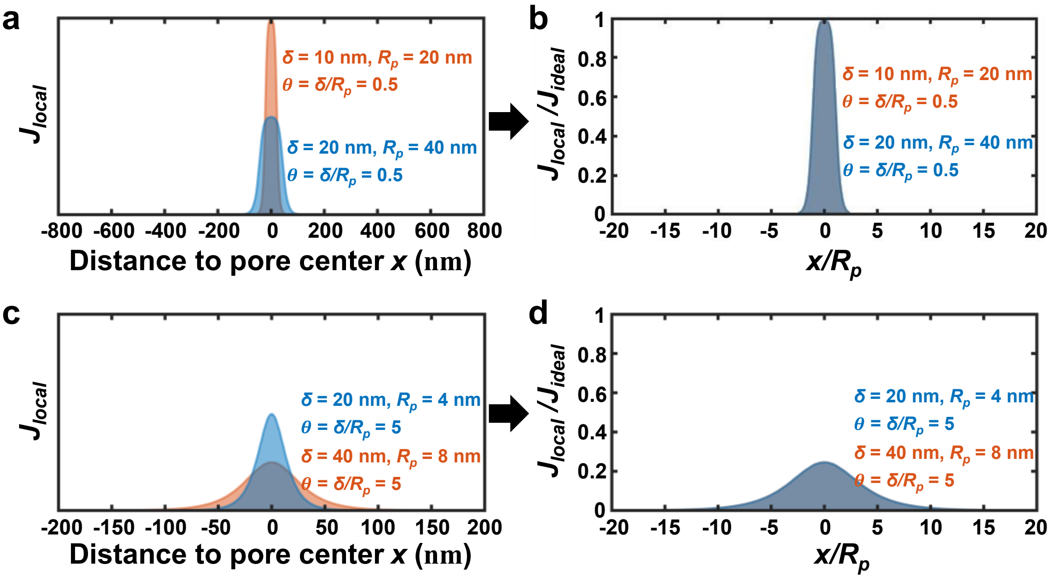


**Figure S4.** (a,c) Local water flux distributions ($J_{local}$) as a function of lateral distance to pore center ($x$) . (b,d) Normalized local flux distributions ($J_{local}/J_{ideal}$) as a function of normalized lateral distance to pore center ($x/R_p$). The simulated membrane cells are assumed to be infinitely large in the lateral dimension and contain a single substrate pore.

## S5. Water transport streamlines in the PA layer

When $\theta$ = 0.5, as water transport from the outer surface to the pore opening, the streamlines entering the film from the top pore region (blue lines) are relatively straight while those from outside the top pore region (purple lines) tend to concentrate near the edge of the pore opening (**Figure S5a**). As the film thickness increases, for example, when $\theta$ = 5 (**Figure S5d**), the purple streamlines will occupy more pore opening area when exiting the film, which squeezes the available area for the transport of blue streamlines.

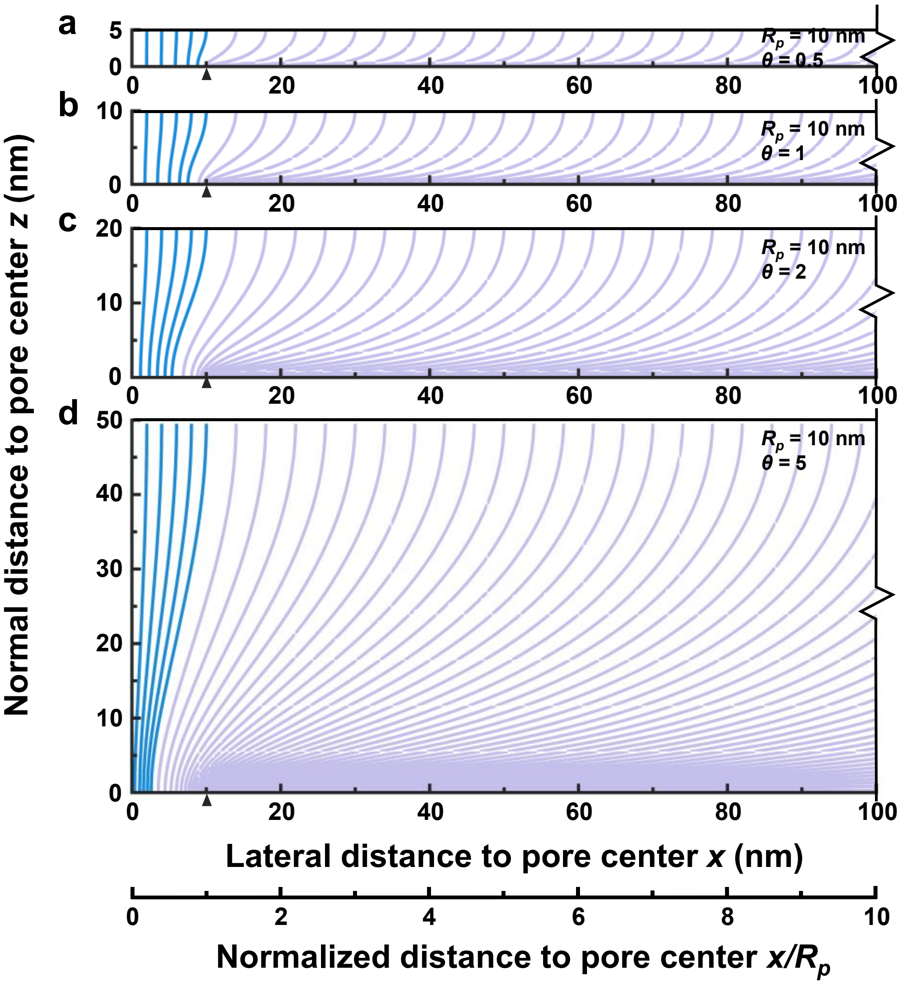


**Figure S5.** Diffusion streamlines in the PA layer for infinite membrane cells. The PA thickness ($\delta$) varies from 5 nm to 50 nm, and the substrate pore size ($R_p$) is fixed at 10 nm. The location of $x$ = 0 represents the substrate pore center, and the location of $x$ = 10 nm represents the pore edge (indicated by the black triangle). The blue streamlines enter the film right above the substrate pore region while the purple

streamlines are from outside the pore region. The starting points of blue and purple streamlines do not change with the PA layer thickness.

## S6. Effect of substrate pore size on water transport patterns

**Figure S6** shows the influence of substrate pore size on water transport patterns across the PA layer. When the substrate pore size is relatively large (e.g., $R_p$ = 40 nm in **Figure S6a**), the water transport streamlines are highly concentrated near the pore region and follow nearly straight pathways in the normal direction within the pore region. With decreasing substrate pore size, the water transport streamlines tend to disperse to encompass a much wider area compared to the substrate pore size.

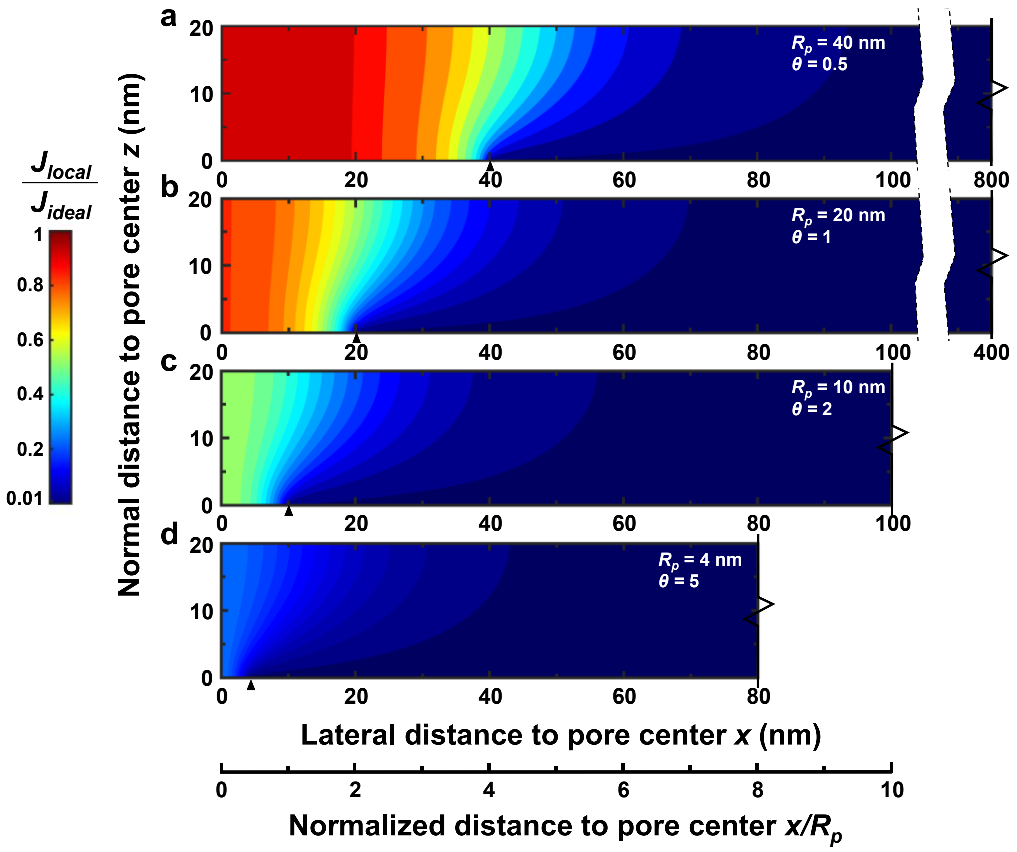


**Figure S6.** Effect of substrate pore size on water transport patterns for unit membrane cells with infinite lateral dimensions and a single substrate pore. The substrate pore size $R_p$ varies from 4 nm to 40 nm and the PA thickness is fixed at 20 nm. The location of $x$ = 0 represents the substrate pore center, and the pore edge is indicated by the black triangle.

## S7. Flux behaviors of thin and thick membranes

**Figure S7a** shows that for thin film ($\theta < 1$), $J_{local}/J_{ideal}$ will approach 1 at the pore center ($x/R_p = 0$) and 0.7 at the pore edge ($x/R_p = 1$), respectively. As the film thickness increases ($\theta > 1$), $J_{local}/J_{ideal}$ at these locations will gradually decrease. In contrast, at the location far from the substrate pore (e.g., $x/R_p = 5$), $J_{local}/J_{ideal}$ will approach 0 when $\theta < 1$ and will increase as the film thickness increases. Accordingly, the PA layer spanning directly over the pore region becomes increasingly responsible for water permeation with decreasing thickness of the PA layer (**Figure S7b**). In **Figure S7c**, if we plot the local flux normalized by the maximum flux ($J_{local}/J_{max}$) against the normalized distance to the pore edge ($(x\text{-}R_p)/\delta$), thin films have overlapping water flux distributions regardless of the $\theta$ value. Similarly, flux distributions of thick films with different $\theta$ values also almost overlap, which has major implications for flux prediction.

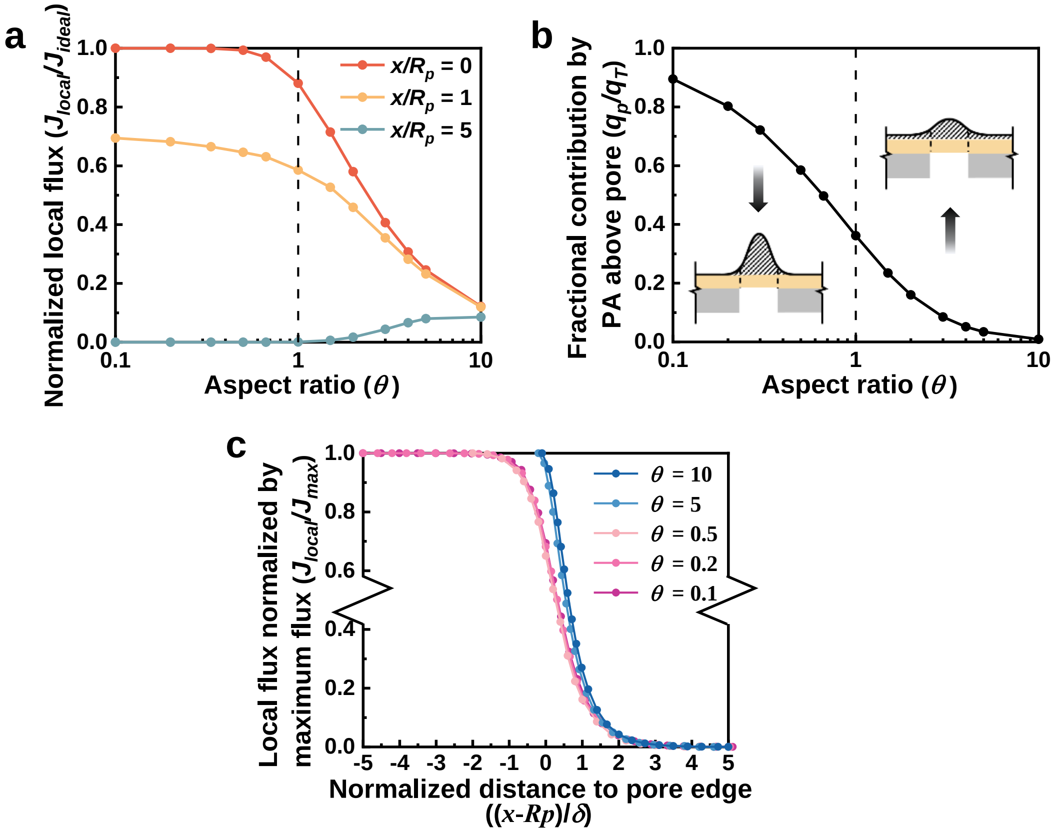


**Figure S7.** The influence of aspect ratio ($\theta$) on (a) the normalized local flux ($J_{local}/J_{ideal}$) and (b) fractional contribution by the PA directly above the pore region ($q_p$) relative to the total flow rate of a single pore ($q_T$). Additionally, two schematic drawings are superimposed to depict the difference in the fractional

contribution for thin and thick films. (c) The distribution of local water flux normalized against the maximum flux ($J_{local}/J_{max}$) as a function of normalized distance to pore edge ($(x\text{-}R_p)/\delta$). $J_{max}$ is the maximum local water flux which occurs right above the pore center. The simulated membrane cells are assumed to be infinitely large in the lateral dimension and contain a single substrate pore.

## S8. Effect of aspect ratio on zone of influence

**Figure S8** presents color contours to illustrate the effect of aspect ratio ($\theta$) on the normalized zone of influence (ZOI) and normalized average zone of influence ($\overline{\text{ZOI}}$). The $\overline{\text{ZOI}}$ based on $J_{ave}/J_{ideal}$ is generally wider than the ZOI based on $J_{local}/J_{ideal}$. As $\theta$ increases, the ZOI and $\overline{\text{ZOI}}$ generally first expand and then narrow, which can be attributed to the competition between the reduction in the head local fluxes and the increase in the tail local fluxes over the top membrane surface.

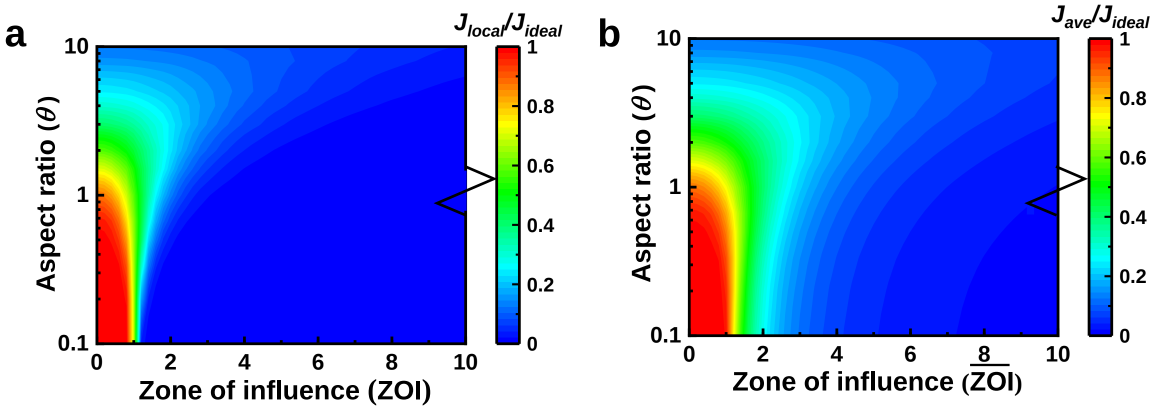


**Figure S8.** Color contour maps of (a) normalized local flux ($J_{local}/J_{ideal}$) and (b) normalized average flux ($J_{ave}/J_{ideal}$). The aspect ratio $\theta$ varies from 0.1 to 10. The simulated membrane cells are assumed to be infinitely large in the lateral dimension and contain a single substrate pore with a fixed pore size of 10 nm.

## S9. Characteristic slant angles based on the average zone of influence

Based on $J_{ave}/J_{ideal}$, we could measure $\overline{\mathrm{ZOI}}$ corresponding to different threshold values $\bar{\xi}$. Following the approach used for ZOI analysis in **Figure 4**, we can obtain the characteristic slant angles $\bar{\phi}$ based on $\overline{\mathrm{ZOI}}$ in **Figure S9.** If a membrane is designed to have a minimum overall water permeation efficiency of 0.5 (i.e., $\bar{\xi}$ = 0.5), we can determine $\bar{\phi}$ from **Figure S9** and identify $\overline{\mathrm{ZOI}_{0.5}}$ region based on the $\bar{\phi}$ angle. The substrate pore distribution could be optimized accordingly until a complete coverage of $\overline{\mathrm{ZOI}_{0.5}}$ region across the PA surface.

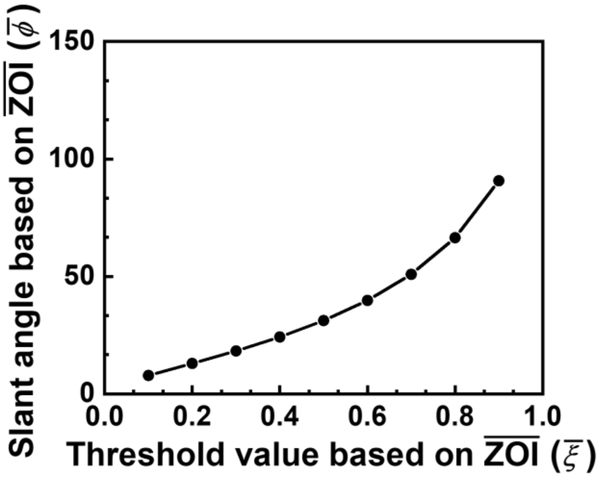


**Figure S9.** The correlation between the slant angle $\bar{\phi}$ and the threshold value of water permeation efficiency $\bar{\xi}$ based on $\overline{\mathrm{ZOI}}$ for thin films featuring $\theta \leq 0.5$. Membranes with different PA thicknesses and a fixed substrate pore size $R_p$ of 10 nm are simulated.

## S10. Accuracy test of superposition principle

In **Figure S10a**, we simulated the total flow rate for a substrate containing two substrate pores ($Q_T$). At the same time, we multiplied the flow rate of each pore ($q_T$) by the number of pores ($N$ = 2). To test the superposition principle, we compared $Q_T$ and $2q_T$ at different aspect ratios in **Figure S10a**. For thin membranes with a low aspect ratio ($\theta \leq 0.5$), the superposition principle works well regardless of the pore-to-pore distance, which could be proved by the flow rate ratio ($Q_T/2q_T$) close to 1. However, the flow rate ratio will slightly decrease for thick membranes ($\theta >> 1$) especially when the two substrate pores are close to each other. Since this study primarily focuses on thin films, the superposition principle could work with reasonable accuracy even when the number of substrate pores ($N$) increases (**Figure S10b**).

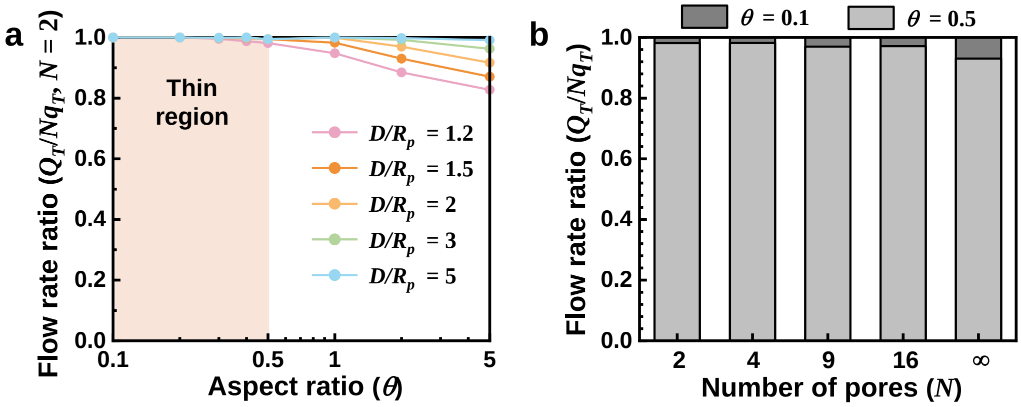


**Figure S10.** Sensitivity test of superposition principle (a) in the presence of two substrate pores considering different pore-to-pore distance (where $D$ represents half of the pore-to-pore distance) and (b) for thin films with different substrate pore numbers at a fixed pore-to-pore distance ($D/R_p$ = 1.2). The simulated membrane cells are assumed to be infinitely large in the lateral dimension and contain a single substrate pore with a fixed pore size ($R_p$) of 10 nm.

**S11. Total flow rate calculation of a multi-pore system**

Based on the superposition principle, the total flow rate ($Q_T$) of a multi-pore system can be expressed as a product of the number of pores ($N$) and the total flow rate of a single pore ($q_T$):

$$Q_T = N \cdot q_T \tag{S1}$$

Here we introduce a flow rate enhancement factor $\gamma$ as follows:

$$\gamma = \cdot q_T / q_{ideal} \tag{S2}$$

$$q_{ideal} = J_{ideal} \cdot S_p \tag{S3}$$

where $S_p$ is the area of the substrate pore region, $J_{ideal}$ is the ideal water flux of a free-standing polyamide film and their product is denoted as the ideal flow rate over the pore region ($q_{ideal}$). As a result,

$$Q_T = N \cdot q_T = N \cdot \gamma \cdot q_{ideal} = N \cdot \gamma \cdot J_{ideal} \cdot S_p = \gamma \cdot \varepsilon \cdot J_{ideal} \cdot S_{PA} \tag{S4}$$

where $\varepsilon$ is the substrate porosity and $S_{PA}$ is the area of PA surface. By some transformation, we can get:

$$\frac{Q_T}{J_{ideal} \cdot S_{PA}} = \frac{J_{app} \cdot S_{PA}}{J_{ideal} \cdot S_{PA}} = \frac{J_{app}}{J_{ideal}} = \frac{A_{app}}{A_{ideal}} = \gamma \cdot \varepsilon \tag{S5}$$

Here, $J_{app}$ and $A_{app}$ are the apparent water flux and water permeance of a substrate-supported polyamide membrane, whereas $J_{ideal}$ and $A_{ideal}$ are the corresponding values of a free-standing polyamide film.

## S12. Sensitivity analysis of a multi-pore system

**Figure S11** illustrates the effect of film thickness $\delta$ and substrate porosity $\varepsilon$ on the water permeation efficiency $A_{app}/A_{ideal}$ for a multi-pore system. The proposed analytical model generally shows a good agreement with COMSOL simulations except for two cases: (1) $R_p$ = 10 nm and $\delta$ = 50 nm in **Figure S11a**, and (2) $R_p$ = 10 nm and $\delta$ = 10 nm in **Figure S11b**. These two cases exhibit relatively high $\theta$ values of 5 and 1, respectively.

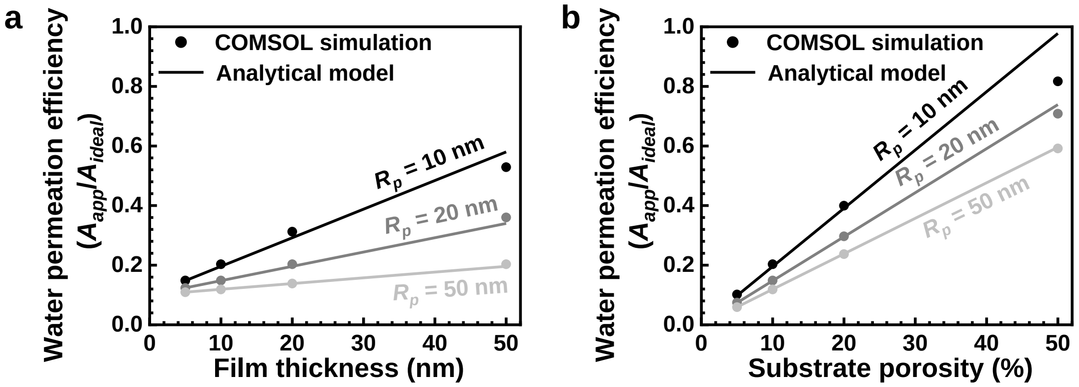


**Figure S11.** The influence of (a) film thickness $\delta$ and (b) substrate porosity $\varepsilon$ on the water permeation efficiency $A_{app}/A_{ideal}$ for a multi-pore system considering different pore sizes. In (a), the film thickness varies from 5 nm to 50 nm at a fixed substrate porosity of 10%. In (b), the substrate porosity varies from 5% to 50% at a fixed film thickness of 10 nm.

**References:**

(1) Jiang, C.; Zhang, L.; Li, P.; Sun, H.; Hou, Y.; Niu, Q. J. Ultrathin Film Composite Membranes Fabricated by Novel In Situ Free Interfacial Polymerization for Desalination. *ACS Appl. Mater. Interfaces* **2020**, *12* (22), 25304-25315.
